# Differences in the body image based on physical parameters among young women from the Czech Republic and Slovakia

**DOI:** 10.1093/eurpub/ckae082

**Published:** 2024-05-11

**Authors:** Ramona Babosová, Barbora Matejovičová, Vladimír Langraf, Miroslav Kopecký, Anna Sandanusová, Kornélia Petrovičová, Janka Schlarmannová

**Affiliations:** Department of Zoology and Anthropology, Constantine the Philosopher University in Nitra, Nitra, Slovakia; Department of Zoology and Anthropology, Constantine the Philosopher University in Nitra, Nitra, Slovakia; Department of Zoology and Anthropology, Constantine the Philosopher University in Nitra, Nitra, Slovakia; Institute of Preclinical Sciences, Palacky University in Olomouc, Olomouc, Czech Republic; Department of Zoology and Anthropology, Constantine the Philosopher University in Nitra, Nitra, Slovakia; Institute of Plant and Environmental Sciences, University of Agriculture in Nitra, Nitra, Slovakia; Department of Zoology and Anthropology, Constantine the Philosopher University in Nitra, Nitra, Slovakia

## Abstract

**Background:**

The increased prevalence of overweight and obesity in the Czech Republic and Slovakia has led to heightened emphasis on weight control, particularly among women. Our aim is to explore body image perceptions among women in both countries and compare their attitudes, focusing on the relationship between body image and body mass index (BMI), height, weight, body fat and the weight control.

**Methods:**

The cross-sectional study involved 358 female students from the University of Pardubice and Constantine the Philosopher University in Nitra, with equal representation from the Czech Republic and Slovakia. Body parameters were assessed using anthropometric methods and the InBody 230 diagnostic device, while participants’ perceptions of their bodies were evaluated using the Body Shape Questionnaire.

**Results:**

The results confirmed that BMI did not significantly influence self-perception among Slovak students, while Czech participants with increasing obesity tended to perceive themselves more negatively. Significantly higher median values for BMI (*p* = 0.0509), weight (*p* = 0.0507), height (*p* = 0.05) and body image (*p* = 0.002) were observed in the Czech Republic compared with Slovakia. No significant difference was found in weight control and fat between participants from both countries.

**Conclusions:**

Although obesity was demonstrated in both nations, body satisfaction was different for participants from the Czech Republic and Slovakia.

## Introduction

The Czech Republic and Slovakia are two neighbouring Central European countries that were part of Czechoslovakia until its dissolution in 1993. In addition to a shared history, both countries have many common elements in gastronomy, such as dumplings, pasta, cabbage soup and fried cheese. Both nations take pleasure in traditional dishes and beverages.

Due to the increasing prevalence of overweight and obesity in both countries, weight control has become more important among Czech and Slovak women in recent years. According to a 2018 study by the World Health Organisation (WHO), the prevalence of female obesity in 2016 was 25.4% in the Czech Republic and 19.9% in Slovakia. The study also revealed a significant increase in the prevalence of female obesity in both countries over the period from 1997 to 2016. In the Czech Republic, obesity rates rose from 21.9% to 25.4%, while in Slovakia they increased from 15.8% to 19.9%.^1^

As indicated by several studies,^2–5^ the prevalence of overweight and obesity among Czech and Slovak women poses a significant problem, entailing associated direct and indirect costs such as healthcare expenditure, loss of productivity and impact on quality of life. In addition to the impact on physical health, perceptions of body weight and dissatisfaction with one’s own body are also important factors to consider.

Weight, body fat percentage, height and the weight control play crucial roles in determining body composition and overall health. In the context of health and well-being, discussion often revolves around body image and body mass index (BMI). Although they are related, they have different meanings and consequences.

Body image refers to the subjective perception and evaluation of one’s own body, including its size, shape and appearance. It plays a crucial role in the development of self-esteem and general well-being. It is influenced by various factors, for example social norms, representations in the media, cultural norms and personal experiences. Body image can be divided into positive body image (PBI) and negative body image (NBI).^6^ PBI involves a healthy and accepting attitude towards one’s body, while NBI involves dissatisfaction, criticism and negative feelings towards one’s body.^7^

The media, in particular, play an important role in shaping body perceptions and ideals. Exposure to media images depicting the slimness ideal has been linked to body image disorders, especially in women.^6^ In recent years, the focus has increasingly shifted to promoting PBI and challenging societal ideals of beauty. The body positivity movement, especially on social media platforms such as Instagram, aims to challenge prevailing beauty standards and promote acceptance and appreciation of different body types and appearances. Content analysis of body-positive posts on Instagram has revealed themes of self-acceptance, body diversity and self-empowerment.^8^^,^^9^

BMI is a numerical value calculated based on a person’s weight (in kilograms) divided by the square of their height (in meters). While it is commonly used as an indicator of weight status, it is not a direct measure of body fat percentage. BMI is a useful tool for assessing weight status at a population level, but has its limitations when applied to individuals as it does not take into account factors such as muscle mass and body fat distribution.^7^ Individuals with a higher BMI may have higher muscle mass and be physically fit rather than have excess body fat.^10^

It is important to note that body image does not always correspond to BMI. Studies have found that people can have a distorted perception of their body image, with some underestimating their weight status and others overestimating it.^11^ These findings suggest that individuals may have a distorted perception of their body image, leading to inaccurate assessments of their BMI. Research has shown that body weight perception, which is part of body image, can be a better predictor of weight management behaviours than actual weight status (BMI).^12^ Furthermore, it is important to note that while BMI provides insight into body composition, it does not encompass all aspects of health.^13^

Our study aims to investigate the perceptions of body image among the women of the Czech Republic and Slovakia and explore potential similarities or differences in their attitudes toward their bodies. Therefore, in our study we (i) analyzed the relationship between body image and BMI, height, weight, body fat and weight control in female students from the Czech Republic and Slovakia separately and (ii) compared the observed variables between female students from the Czech Republic and Slovakia.

## Methods

Our cross-sectional study was conducted on 358 female students from the University of Pardubice and Constantine the Philosopher University in Nitra [179 (50%) from the Czech Republic and 179 (50%) from Slovakia]. Female students were between 18 and 24 years of age, with an average age of 20 years for the Slovak and Czech subject populations. All participants were informed of the purpose of the study, and written informed consent was obtained from them. As this was a voluntary and anonymous study that did not affect the dignity of the individual, no evaluation by an ethics committee was required.

To assess participants’ perceptions of their own bodies, we used the Body Shape Questionnaire, in which respondents expressed their subjective feelings about their bodies. This scale was originally created by Cooper et al.,^14^ with a one-factor structure consisting of 34 self-report items and using a 4-point Likert-type response scale. In our study, the questionnaire was designed based on the abbreviated 8-item version by Evans and Dolan.^15^

Participants can express their body image on a 4-point scale (ranging from 0 ‘not at all’ to 3 ‘very much’). The total score (score point), ranging from 0 to 24, is obtained by summing up the scores of the 8 items. A higher score indicates a higher level of body image disturbance. The Cronbach’s alpha for reliability in our study was 0.73, which we calculated in the program Statsoft, INC. (2004).^16^

The questionnaire began with a demographic section and included quality of life ratings related to weight, binge eating, body image and body shame. Questions were closed-ended, and participants had the option to select a response: agree, disagree, or I don't know, I'm not aware. Subsequent statistical analysis included scoring of participants' responses to each question.

Body height (cm) was measured using the standard anthropometric method with the A-226 anthropometer.^17^ For each participant, body weight (kg), fat mass and BMI (kg/m^2^) were measured using the bioimpedance method with one the InBody 230 diagnostic device, purchased in 2017 (Inbody 230, Biospace Corp., Seoul, Korea) based on the InBody 720.^18^ Calibration was verified before measurements in each country.

According to the manufacturer, the InBody 230 is suitable for people aged 3–99. InBody 230 generates 10 impedance values with two different frequencies to measure five body segments (right leg, left leg, right arm, left arm and boot). Body composition estimates were calculated using the manufacturer's software (Lookin’Body 120, Biospace Corp., Seoul, Korea). According to the Lookin'Body software of the InBody 230 device, we considered 18.1–28% as the normal range for body fat percentage in women. This range is relatively narrow, but it is based on the fact that the participants are young adults, and it is known that body fat percentage tends to increase with age.

Weight control provides participants with guidance to adjust their diet and exercise to achieve a healthy percentage of body fat. These data are useful for monitoring the health of the participants. Depending on the current muscle-fat balance of the subject, the weight control value determines whether an adjustment to fat and/or muscle mass is recommended. The symbol ‘+’ indicates the goal to gain, while ‘-’ implies the objective to lose body fat mass. If the subject is overweight, InBody advises on losing a certain amount of fat mass while maintaining or increasing muscle mass. InBody never recommends losing muscle mass.

The statistical program R. 6.3.6^19^ was used for analysis focused on linear regression, which we used to determine the dependence between body image (score points) and BMI (kg/m^2^), height (cm), weight (kg), fat (kg) and weight control (kg) separately in probands from the Czech Republic and Slovakia. The dependent variable is body image (score points), the other variables are independent. The Kolmogorov–Smirnov test was used to determine the normality of the data distribution of the studied variables. Due to the violation of normality data distribution, the non-parametric Mann–Whitney *U* test was used to test for differences in variables (body image, BMI, height, weight, fat, weight control) in participants from the Czech Republic and Slovakia, as well as between countries (Czech Republic, Slovakia) each other. This test was employed to examine differences between the two selected independent populations of respondents (Czech Republic and Slovakia). We performed a basic statistical description of the studied variables using descriptive statistics.

## Results

The distribution of weight categories among participants from the Czech Republic and Slovakia was similar, with the majority falling into the healthy weight range, followed by overweight, underweight, and the smallest number in the obese category. In total, out of 358 participants, 246 were in the healthy weight range, 58 were overweight, 35 were underweight and 19 were obese (Supplementary table S1).

We used descriptive statistics to provide a basic statistical description of the investigated variables body image, BMI, height, weight, fat, weight control, for separate populations of respondents in the Czech Republic and Slovakia (table 1). The results of the analysis show that Czech respondents had higher average, median and minimum values compared with Slovak respondents. The opposite was true for most of the maximum and standard deviation.

**Table 1:** Basic statistical description of the investigated variables for respondents from the Czech Republic and Slovakia

Nationality	Variables	Mean	Median	Minimum	Maximum	Standard deviation
Czech	Body image (score)	62.81	63.00	42.00	101.00	8.49
Slovak		59.04	60.00	25.00	106.00	11.21
Czech	BMI (kg/m^2^)	22.55	23.00	17.30	32.90	3.23
Slovak		22.36	21.00	16.00	33.40	3.66
Czech	Height (cm)	166.23	168.00	152.30	186.40	6.48
Slovak		164.83	165.00	148.90	183.50	6.31
Czech	Fat (kg)	19.19	17.10	8.70	46.70	7.42
Slovak		19.08	17.50	4.90	44.80	7.77
Czech	Weight (kg)	62.37	62.00	41.00	97.10	10.02
Slovak		60.77	59.00	42.10	95.20	10.50
Czech	Weight control (kg)	−2.43	−0.40	−31.70	11.00	8.38
Slovak		−1.87	−0.20	−29.80	16.10	9.33

**Figure 1:**
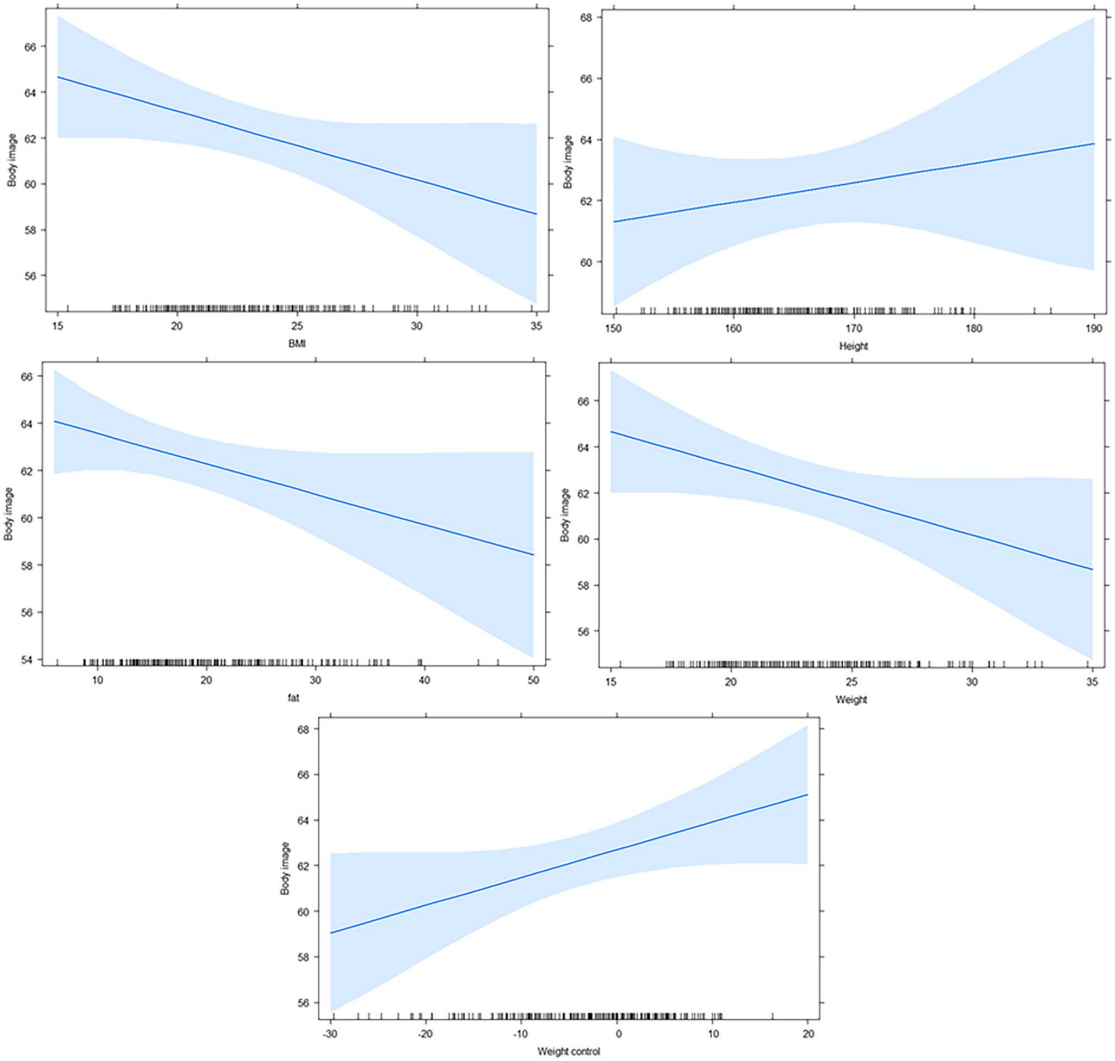
Regression models expressing the dependence between body image and BMI, weight, fat, height and weight control in probands from the Czech Republic

Among the participants from the studied countries, differences in questionnaire scores were tested BI, BMI, height, weight, fat and weight control using the Mann–Whitney *U* test. Due to violation of the normality data distribution for body image (score points) (*P* < 0.001), BMI (*P* < 0.001), height (cm) (*P* = 0.03909), weight (kg) (*P* < 0.001), weight control (kg) (*P* < 0.001), fat (kg) (*P* < 0.001) we used non-parametric Mann–Whitney *U* test. We confirmed a statistically significant difference between Czech Republic and Slovakia for the variables body image (*P* = 0.002, *Z* = 3.0866, *U* = 13000.5, median for Czech Republic = 63, median for Slovakia = 60), height (cm) [*P* = 0.05, *Z* = 1.8967, *U* = 14 163.5, median for Czech Republic = 168 (cm), median for Slovakia = 165 (cm)], weight (kg) [*P* = 0.0507, *Z* = 1.8899, *U* = 14170, median for Czech Republic = 62 (kg), median for Slovakia = 59 (kg)], BMI (*P* = 0.0509, *Z* = 1.8888, *U* = 14 171, median for Czech Republic = 23, median for Slovakia = 21). For BMI, weight, height and BI, median values were higher in the Czech Republic than in Slovakia. The median value for weight control and fat was higher in Slovakia. No significant difference was found for weight control (kg) (*P* = 0.5, *Z* = −0.6736, *U* = 15 361, median for Czech Republic = −0.40, median for Slovakia = −0.20) and fat (kg) (*p* = *P* = 0.9, *Z* = 0.1092, *U* = 1591.5, median for Czech Republic = 17.10, median for Slovakia = 17.40) (figure 1).

**Figure 2:**
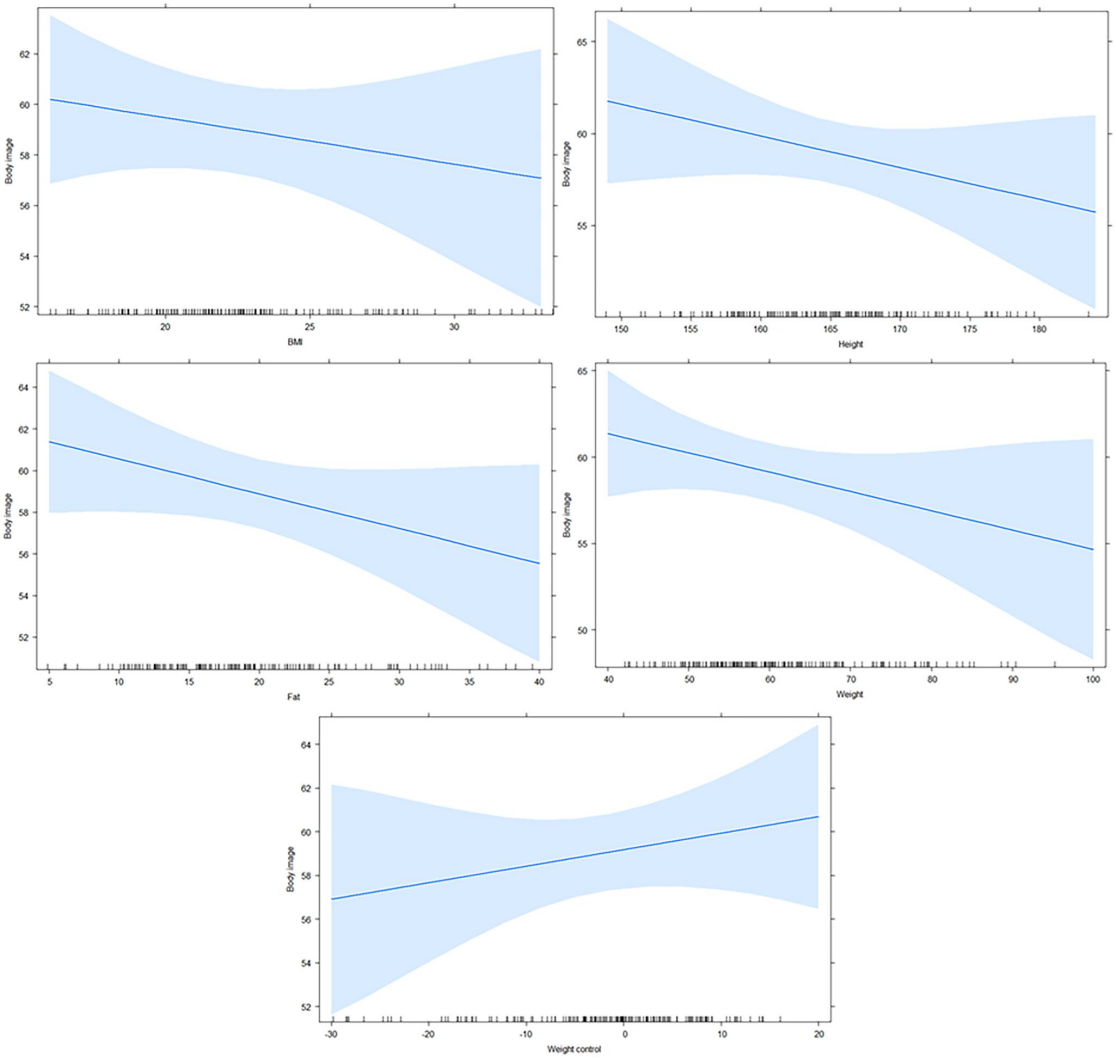
Regression models expressing the dependence between body image and BMI, weight, fat, height and weight control in probands from Slovakia

In the participants’ studies, a regression model was used to show the dependence between body image and BMI, height, weight, fat and weight control in participants from the Czech Republic and Slovakia separately. In Czech participants, a trivial negative dependence was found between body image and BMI (*r* = −0.1671), weight (*r* = −0.151), and fat (*r* = −0.1678). Thus, BMI, weight and fat had a negative effect on the participants' perception of their appearance, and their increasing values increased their dissatisfaction with their own perception. Moderate dependence was found for height (*r* = 0.4674) and weight control (*r* = 0.4802) (figure 2).

The taller people were, the more weight they were advised to gain, the higher their self-satisfaction. The coefficient of determination expressed the variability between body image and BMI 46.78% (*r*^2^ = 0.4678), height 67.83% (*r*^2^ = 0.6783), weight 46.78% (*r*^2^ = 0.4678), fat 67.02% (*r*^2^ = 0.6702) and weight control 61.32% (*r*^2^ = 0.6132). The models confirmed a significant linear dependence between body image and BMI (*P* = 0.0362, *F*-statistic = 0.8318, *t*-statistic = −0.912), height (*P* = 0.0491, *F*-statistic = 0.4753, *t*-statistic = −0.6894), fat (*P* = 0.0420, *F*-statistic = 0.6520, *t*-statistic = −0.8074), weight (*P* = 0.0063, *F*-statistic = 0.2264, *t*-statistic = −0.4758), and weight control (*P* = 0.0297, *F*-statistic = 1.0922, *t*-statistic = 1.0451).

From the total sum of squares, our body image and BMI model expressed 12 772 squares, of which 60 are residues. Height expressed 12 797 squares, of which 72 were residues. Weight expressed 12 815 squares, of which 72 were residues. Fat expressed 12 784 squares, of which 72 were residues and weight control 12 752 squares, of which 72 were residues.

**Figure 3:**
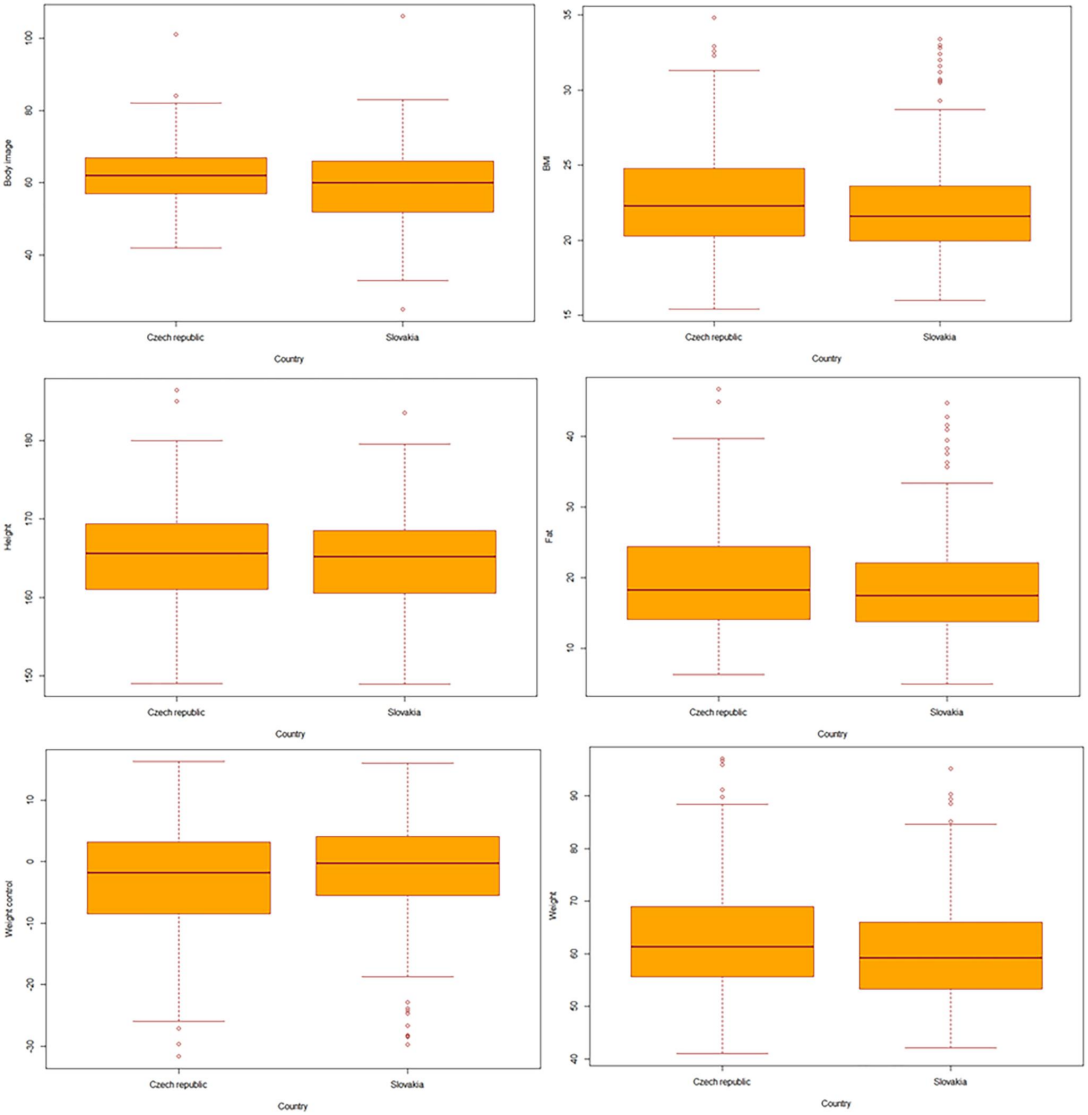
Differences in the examined variables (body image, BMI, weight, fat, height, weight control) between countries

For the Slovak students, linear regression confirmed a trivial negative dependence between body image and weight (*r* = −0.1015) and fat (*r* = −0.1165). Thus, weight and fat had a negative effect on the participants' perception of their appearance, and their increasing values increased their dissatisfaction with their own perception. The values for BMI (*r* = −0.0927) and height (*r* = −0.0707) were negative but very close to zero, indicating zero dependence, that is, BMI and height had no effect on satisfaction with self-perception. Weight control had a value of *r* = 0.0875, close to zero indicating zero dependence and weight control did not influence self-perception satisfaction (figure 3).

The coefficient of determination expressed the variability between body image and BMI 60.69% (*r*^2^ = 0.6069), height 94.33% (*r*^2^ = 0.9433), weight 56.65% (*r*^2^ = 0.5665), with fat 88.08% (*r*^2^ = 0.8808) and with weight control 68.72% (*r*^2^ = 0.6872). The regression models confirmed statistically significant linear relationships between body image and BMI (*P* = 0.04245, *F*-statistic = 0.6407, *t*-statistic = −0.8004), height (*P* = 0.0195, *F*-statistic = 1.6856, *t*-statistic = −1.2983), weight (*P* = 0.0163, *F*-statistic = 1.9608, *t*-statistic = −1.4002), fat (*P* = 0.0123, *F*-statistic = 2.4018, *t*-statistic = −1.5497), and weight control (*P* = 0.0402, *F*-statistic = 0.7052, *t*-statistic = 0.8398).

From the total sum of squares, our body image and BMI model expressed 22 294 squares, of which 125 were residues. Height expressed 22 163 squares, of which 125 were residues. Weight expressed 22 129 squares, of which 125 were residues. Fat expressed 22 075 squares, of which 124 were residues, and weight control 22 285 squares, of which 125 were residues.

## Discussion

The study investigated differences in body image, BMI, height, weight, fat, and weight control among young women in Slovakia and the Czech Republic. The results revealed several noteworthy findings that clarified the relationship between these parameters, especially body image and BMI, in these two neighbouring countries.

One of the key findings was a significant difference in BMI values between the two countries. The Czech Republic exhibited significantly higher median BMI values (*P* = 0.0509, median for Czech Republic = 23, median for Slovakia = 21) compared with Slovakia.

This difference may be influenced by various factors, including dietary habits, levels of physical activity and societal norms related to body size and weight. Čuta et al. studied normal-weight obesity in a sample of Czech women and found that depending on the chosen threshold, up to 14% of women were classified as obese with average weight.^20^ In our study, 46.8% of participants were classified as obese with average weight in the Czech Republic, and 48.4% in Slovakia. That suggests that relying solely on BMI may not accurately identify individuals with excess body fat.

Differences in body image scores between Slovakia and the Czech Republic also indicate variations in how young women perceive their bodies. The higher average body image score observed in the Czech Republic suggests that body image concerns may be more prevalent or pronounced in this country. Societal ideals and media representations of beauty often shape individuals’ body perceptions, and prevailing cultural influences in the Czech Republic might contribute to this discrepancy. Body dissatisfaction or NBI is a common issue among young women, with studies indicating that many experience feelings of inadequacy and dissatisfaction with their bodies.^21^^,^^22^ This dissatisfaction can lead to various psychological problems, including anxiety, depression and eating disorders.

Moreover, weight status and body composition can influence body image perception. Studies have shown that individuals who are overweight or obese may be more likely to experience body image dissatisfaction.^23^ Among adolescent girls, there is a link between weight status and body image perception. Therefore, differences in weight status and body composition between the Slovak and Czech samples could have contributed to the observed deviations in the relationships between BMI, height, and body image perception.^23^^,^^24^

A positive correlation between height and weight control with body image in the Czech Republic suggests that taller individuals may be more satisfied with their bodies and have a greater tendency to maintain their weight according to perceived ideals. This finding resonates with studies indicating that taller individuals are often perceived as more attractive and face fewer weight-related stigmas.^25^ On the other hand, in Slovakia, BMI, height and weight control showed negligible correlations with body image. This result suggests that factors beyond physical attributes may significantly shape body perceptions among young Slovak women. Societal norms, and personal experiences are likely to play significant roles in shaping their perceptions of their bodies. Similarly, Tenkorang and Okyere examined factors influencing body image perceptions among university students in Ghana. The study identified societal and cultural backgrounds, family members, peers and media as influential factors in body image perception.^26^ On the other hand, rational dietary habits should be promoted as a component of a healthy lifestyle.^27^

In the case of Slovak overweight female students, no relationship was found between BMI and body dissatisfaction as the BMI value (*r* = −0.0927) was negative but very close to zero, indicating zero dependence, that is, BMI had no effect on satisfaction with self-perception. This finding aligns with body image theories, which suggest that there may be a small relationship between what a person thinks about their body and the objective reality of their appearance. It is assumed that PBI may be related to body positivity, self-empowerment, inclusivity and encouraging individuals to be proud and accepting of their bodies, despite having a BMI that classifies them as clinically overweight/obese.^8^ Self-esteem could be a potential mitigating factor in the adverse association between individuals' BMI, their feelings about their bodies, and the level of anxiety caused by negative evaluations from others.^28^

The coefficient of determination values indicates the extent to which BMI, height, weight, fat and weight control account for the variability in body image perception in each country. In Slovakia and the Czech Republic, these factors explained a substantial portion of the variance in body image scores, highlighting their importance in shaping how young women perceive their bodies.

The lack of significant differences in weight control (kg) and fat (kg) between these two countries suggests that weight management practices and fat levels may be relatively consistent among young women in Slovakia and the Czech Republic. However, further research is needed to explore specific factors influencing weight control behaviour and fat distribution in each population.

## Limitation of study

A strength of our study was that body parameters were measured using an anthropometer and the InBody 230 diagnostic device, which determined BMI based on the obtained results. These results were subsequently compared with the participants' responses provided in the questionnaire. In contrast, some studies use self-reported values from participants to calculate BMI and assess body weight status. However, these values can be biased. Research in this area can help identify potential risk factors and barriers to adopting a healthy lifestyle and aid in developing of targeted interventions to improve body image and promote a healthier attitude towards weight and appearance. Future research could provide more information on how various factors (e.g. sociocultural influences, environmental factors) influence it.

Additionally, further studies should explore the sociocultural factors contributing to body image perceptions and weight-related attitudes among young women in Slovakia and the Czech Republic. Longitudinal studies could offer valuable insights into the temporal relationships between these variables and help elucidate the causal pathways underlying the observed associations. Furthermore, incorporating qualitative research methods such as interviews or focus groups could provide a deeper understanding of the subjective experiences and societal influences shaping body image perceptions in these populations.

## Conclusion

In conclusion, this study provides valuable insights into the differences in body image and BMI among young women in Slovakia and the Czech Republic. A priority in public health efforts in both countries should be the promotion of PBI and healthy weight regulation practices. By understanding the unique challenges faced by young women in Slovakia and the Czech Republic, we can develop targeted interventions that foster body positivity, improve body image satisfaction, and enhance overall well-being in these populations. Further research in this area is essential to address the complex relationship between body image, BMI and cultural influences and to develop comprehensive approaches to effectively tackle obesity and body image issues.

## Supplementary Material

ckae082_Supplementary_Data

## Data Availability

Because of data-sharing restrictions, files containing anonymized datasets cannot be made publicly available. The data will be shared upon reasonable request to the corresponding author. Key points Differences in self-perception and body parameters: The study highlights significant differences between Czech and Slovak female students regarding body mass index (BMI), weight, height and body image perception. Czech participants generally exhibited higher values in these parameters compared with Slovak participants. Impact of obesity on self-perception: The study indicates that increasing obesity among Czech participants correlates with a more negative self-perception. However, BMI did not significantly influence self-perception among Slovak students. Methodological aspects: Utilizing statistical methods such as the Mann-Whitney *U* test and linear regression provided insights into the statistical differences between the two countries in terms of body parameters and their impact on self-perception.
