# Supplementary material for: Differences in the body image based on physical parameters among young women from the Czech Republic and Slovakia
Source: Eur J Public Health. 2024 May 11;34(4):730–6. doi: 10.1093/eurpub/ckae082 (PMC11299200; doi:10.1093/eurpub/ckae082)
Supplement: ckae082_Supplementary_Data [file ckae082_supplementary_data.docx]

| **country** | **BMI** | **weight status** | **number of participants** |
| --- | --- | --- | --- |
| Czech Republic | below 18.5 | underweight | 14 |
|  | 18.5 - 24.9 | healthy weight | 124 |
|  | 25.0 - 29.9 | overweight | 32 |
|  | above 30.0 | obese | 9 |
| Slovakia | below 18.5 | underweight | 21 |
|  | 18.5 - 24.9 | healthy weight | 122 |
|  | 25.0 - 29.9 | overweight | 26 |
|  | above 30.0 | obese | 10 |

**Table S1**. The Overview of Distribution of Weight Categories among Participants from the Czech Republic and Slovakia.
